# A prediction model for underestimation of invasive breast cancer after a biopsy diagnosis of ductal carcinoma in situ: based on 2892 biopsies and 589 invasive cancers

**DOI:** 10.1038/s41416-018-0276-6

**Published:** 2018-10-17

**Authors:** Claudia J. C. Meurs, Joost van Rosmalen, Marian B. E. Menke-Pluijmers, Bert P. M. ter Braak, Linda de Munck, Sabine Siesling, Pieter J. Westenend

**Affiliations:** 1 CMAnalyzing, Gounodstraat 16, 6904 HC Zevenaar, The Netherlands; 2 Department of Biostatistics, Erasmus MC, University Medical Center Rotterdam, Wytemaweg 80, 3015 CN Rotterdam, The Netherlands; 3 Department of Surgery, Albert Schweitzer Hospital, PO Box 444, 3300 AK Dordrecht, The Netherlands; 4 Department of Radiology, Albert Schweitzer Hospital, PO Box 444, 3300 AK Dordrecht, The Netherlands; 5 Department of Research, Netherlands Comprehensive Cancer Organisation, PO Box 19079, 3501 DB Utrecht, The Netherlands; 6 Laboratory of Pathology Dordrecht, Karel Lotsyweg 145, 3318 AL Dordrecht, The Netherlands; 7 Regional screening organization South West the Netherlands, Maasstadweg 12, 3079 DZ Rotterdam, The Netherlands

**Keywords:** Risk factors, Interleukins

## Abstract

**Background:**

Patients with a biopsy diagnosis of ductal carcinoma in situ (DCIS) might be diagnosed with invasive breast cancer at excision, a phenomenon known as underestimation. Patients with DCIS are treated based on the risk of underestimation or progression to invasive cancer. The aim of our study was to expand the knowledge on underestimation and to develop a prediction model.

**Methods:**

Population-based data were retrieved from the Dutch Pathology Registry and the Netherlands Cancer Registry for DCIS between January 2011 and June 2012.

**Results:**

Of 2892 DCIS biopsies, 21% were underestimated invasive breast cancers. In multivariable analysis, risk factors were high-grade DCIS (odds ratio (OR) 1.43, 95% confidence interval (CI): 1.05–1.95), a palpable tumour (OR 2.22, 95% CI: 1.76–2.81), a BI-RADS (Breast Imaging Reporting and Data System) score 5 (OR 2.36, 95% CI: 1.80–3.09) and a suspected invasive component at biopsy (OR 3.84, 95% CI: 2.69–5.46). The predicted risk for underestimation ranged from 9.5 to 80.2%, with a median of 14.7%. Of the 596 invasive cancers, 39% had unfavourable features.

**Conclusions:**

The risk for an underestimated diagnosis of invasive breast cancer after a biopsy diagnosis of DCIS is considerable. With our prediction model, the individual risk of underestimation can be calculated based on routinely available preoperatively known risk factors (https://www.evidencio.com/models/show/1074).

## Background

Patients with ductal carcinoma in situ (DCIS) are treated based on the risk of underestimation or progression to invasive cancer. The standard treatment for patients with a biopsy diagnosis of DCIS is wide local excision with radiation or mastectomy. Often a sentinel lymph node (SLN) biopsy is advised for axillary staging.^1,2^ Both the standard treatment and the use of the SLN biopsy can constitute overtreatment. The standard treatment might be disproportionate for screen-detected DCIS patients who have a high chance that the DCIS would not even have been detected during their lifetime.^3^ It has been estimated that between 14 and 53% of DCIS progress into invasive breast cancer.^4,5^

To address overtreatment, phase III trials investigate the safety of active surveillance of DCIS patients at low risk for developing or having invasive breast cancer.^6–11^ Active surveillance is based on the result of the biopsy. By modelling of active surveillance of DCIS patients, the disease-specific cumulative mortality was related to underestimation.^12,13^ Underestimation is the phenomenon that the invasive breast cancer is undetected at preoperative biopsy and only becomes evident after pathological examination of the excision material. The use of the SLN biopsy can also constitute overtreatment. The SLN biopsy is done if a mastectomy is chosen, and also for patients undergoing wide local excision who are at high risk of having an underestimated invasive breast cancer.^1,2^ The reported risk of underestimation varies from 14 to 43%^14,15^, and in a meta-analysis it was estimated to be 25.9% (95% confidence interval (CI): 22.5–29.5)^16^. These rates indicate that many patients will still have the diagnosis of DCIS after examination of the excision material, and thus the SLN biopsy would not have been necessary.

Knowledge on the risk of underestimation is important in selecting high-risk or low-risk patients for treatment or active surveillance. The most frequently reported risk factors for underestimation are DCIS grade and factors found with radiological diagnostic work-up, such as the size of the lesion, mass on mammography or ultrasonography, and the Breast Imaging Reporting and Data System (BI-RADS) score.^14–27^ Furthermore, these studies reported that the risk of underestimation was associated with age, palpability, histologic suspicion of invasion, image guidance method, biopsy device and other factors. An overview of the found risk factors for underestimation is given in Table 1. Based on risk factors, several studies developed prediction models with the purpose to select patients for SLN biopsy^14,17,24,28–30^.

**Table 1:** Results of previous studies on risk factors for underestimation

Variables^a,b^	Significance of potential risk factors, as described in literature^c,d^		
	No	Yes, univariable^e^	Yes, multivariable^f^
Age	15, 16, 18, **22, 24**, 25	**14**, 21	
Detection mode^g^		**14**, 16	
*Palpable*	**22**	16	15, 18, 23/**28**, **24**, 25, 26
Clinical size of mass		18	
*BI-RADS* ^g^	21, 23	**14**, 15, 16	
Maximum size on imaging		16	**22**
Maximum size on mammography^g^		18, 21, 26	**14**, 20, **24**, 25
Maximum size on ultrasonography^g^		23	15, **30**
Maximum size on MRI		26	**30**
Mass on imaging			**14**
Mass on mammography		16, 26	18
Mass on ultrasonography	**30**	26	23/**28**
Visibility on ultrasonography	16		**24**
Type of mammographic abnormality^g^	**14**, 21, **24**	15	25
Calcifications on mammography	23, 26		**30**
Calcifications on ultrasonography			23/**28**
Suspicious findings on ultrasonography or MRI		**30**	
Multicentric		**14**	
Breast density		**14**	
Residual disease on mammogram after biopsy			21
Calcification % removed by CNB		**14**	
Biopsy guidance technique^g^		**14**, 16, **24**, 26	
Biopsy type CNB, VAB	**24**	**16**, 25	15, **22**, 23/**28**
Biopsy needle gauge		14	
Number of cores obtained^g^	**14**, 21	15, 25, 26	
*DCIS grade*	26	16, 23, **24**, 25	**14**, 15, 20
Nuclear grade	26, **30**		**22**
*Suspicious of invasion on biops*y			23/**28**, **24**
Comedo-necrosis	16, 18, 23, 26	**14**, **22**, 25	**30**
Intraductal structure			25
Cibriform	14		**22**
Sclerosing adenosis			**30**
Hormone receptor ER/PR			**22**
Progesterone receptor		**30**	
HER2	**22**		**30**

^**a**^Variables in bold are variables that were analysed in this study

^b^Variables that were analysed but were not statistically significant in any study were: mass on MRI (30), mass on ultrasonography or MRI (30), abnormality on mammography; mass, asymmetry or distortion (23), calcifications on imaging (22), suspicious findings on ultrasonography (30), suspicious findings on MRI (30), solid (14, 22) papillary (14, 22), micropapillary (14, 22), necrosis (22), oestrogen receptor (14,30), period from breast biopsy to surgery (25), Van Nuys grouping (23), family history (21), menopausal status (21) and type of first resection (18)

^c^Listed are 12 studies with at least 100 cases of underestimation

^d^References in bold are of the five studies that developed a prediction model

^e^Reference ^16^ presents results of random-effect logistic regression models in a meta-analysis

^f^The multivariable significant variables of ref. ^23^ were used in a prediction model, as described in ref. ^28^

^g^The categories of these variables were not uniformly defined between studies

Besides the underestimation rate, other factors are useful for making a treatment plan for a patient diagnosed at biopsy with DCIS. First of all, for some of these patients, no residual disease is found in the excision material; this is defined as minimal-volume DCIS. A rate of 9.3% was reported.^31^ Second, of the underestimated invasive breast cancers the information on unfavourable features is of interest; the reported Her2Neu status is quite high^22,23^ and the hormonal receptor statuses vary^21–23,25,26^.

The diversity of identified risk factors for underestimation has resulted in differences between the clinical guidelines used in different countries. For example, according to the NICE guideline (United Kingdom) for the use of the SLN biopsy, risk factors for underestimation are a palpable mass or extensive micro-calcifications, while according to the Dutch guideline, these are age <55 years, intermediate-grade or high-grade DCIS, a mass on mammography and a suspected invasive component based on biopsy. For active surveillance, the main criterion for patient selection in low-risk DCIS trials are DCIS grade, and patients with mass or other relevant factors are excluded.

The diversity in risk factors might be due to the study designs, since the investigated potential risk factors varied and many studies on underestimation were single institution studies with limited number of cases. Information at the population level is lacking. In addition, there is hardly any data on minimal-volume DCIS nor on the presence of unfavourable features of the underestimated invasive breast cancer.

The aim of our study was to expand the knowledge on underestimation of invasive breast cancer for patients with a biopsy diagnosis of DCIS in routine clinical practice in the Netherlands and to develop a prediction model based on population data. We also analysed the association of predicted risk with minimal-volume DCIS and with the occurrence of unfavourable features of the underestimated invasive breast cancer. The results could contribute to a treatment plan that is both patient-specific and helps in reducing overtreatment.

## Methods

### Study design and population

This study used retrospective data that were nationwide. Data were received from the Dutch Pathology Registry, which is managed by PALGA (the nationwide network and registry of histopathology and cytopathology in the Netherlands) and were matched with data from the Netherlands Cancer Registry (NCR), which is hosted by the IKNL (the Netherlands Comprehensive Cancer Organization). The Dutch Pathology Registry contains all the reports written by pathologists of material examined in all Dutch Pathology Laboratories.^32^ The NCR contains information that is collected and coded by specially trained registration clerks from the hospitals’ patient files of every patient with cancer, after notification from PALGA.^33^

Lesions were selected from PALGA, since this study is based on the biopsy diagnosis and the NCR registers the final diagnosis at excision. Histological breast biopsies were selected that were performed in the period 1 January 2011 until 30 June 2012. The diagnosis should be carcinoma in situ, with no invasive cancer in the same biopsy, no lymph node metastases found preoperative and also no melanoma in situ, Morbus Paget or Morbus Bowen. DCIS with micro-invasion was not included, nor were intracystic carcinoma, lobular carcinoma in situ and ductal hyperplasia lesions. Based on the PALGA conclusion (free text field) information on the diagnosis, DCIS grade, suspected invasive component, synchronous contralateral tumour and ipsilateral history were coded. The data were extended with those registered by the NCR: age, ipsilateral history, detection mode, palpability, BI-RADS score, preoperative magnetic resonance imaging (MRI), multidisciplinary team meeting, type of first resection, nodal status, and of the invasive cancers, the morphology, grade, the receptors ER, PR, Her2Neu and tumour size. Lesions were excluded in case of incomplete registration, primarily no excision of the lesion, a biopsy diagnosis that was inconclusive as to whether the lesion was benign or DCIS and in case of an ipsilateral history of DCIS or invasive breast cancer.

Data were categorized as follows: the category detection mode consisted of screen-detected DCIS (DCIS detected within 12 months after a positive mammography at the population-based screening programme) and otherwise detected DCIS (all other DCIS). DCIS grade was categorized into low, intermediate or high. If the tumour consisted of two different grades or if the grade was inconclusive, the highest DCIS grade was chosen. Suspected invasive component was coded ‘yes’ if it was mentioned as such in the conclusion of the pathology report and if it was not refuted with potential additional staining. For the BI-RADS score, no subgroup information for score 4 was available.^34^ A synchronous contralateral lesion was defined as DCIS or invasive breast cancer in both breasts with a difference in incidence date of <3 months. Underestimation was defined as invasive cancer or micro-invasion found at excision after a biopsy diagnosis DCIS. Tumours were graded according to the Bloom–Richardson grade or another equivalent method. Tumour size and nodal status were used to categorize the tumor node metastasis (TNM) stage.^35^ Underestimated invasive breast cancers were categorized based on unfavourable features. In the Dutch guideline^36^, they were defined as features that, if present, would mean that systemic therapy would be recommended, because the absolute 10-year mortality risk was at least 15%. These features of the invasive cancers were:

- Her2Neu positive with size >5 mm.
- Age <35 years, except size ≤10 mm with grade I.
- Size >10 mm but ≤20 mm with grade II or III.
- Size >20 mm.
- Positive lymph nodes.

### Statistical analysis

The data were analysed to investigate associations and to develop a prediction model. First, the distribution of patient characteristics and potential risk factors was compared between patients with and without underestimated invasive breast cancer for the non-missing values, using the Mann–Whitney test or the Pearson's *χ*^2^ test. The associations between potential risk factors were analysed with the Pearson's *χ*^2^ test or the Fisher's exact test. The risk for underestimation of invasive breast cancer was analysed with logistic regression analysis. The threshold for significance of risk factors was the two-sided *p* value of 0.05. In this logistic regression, we only included characteristics that were known as independent variables prior to operation: age, detection mode, palpability, BI-RADS score, DCIS grade and suspected invasive component at biopsy. The decision to do a preoperative MRI and the type of first resection were not included in the model, because no causal association with underestimation was expected. Next, to ensure that all relevant variables were included in the prediction model, the independent variables were chosen via stepwise backward selection with a *p* value threshold for elimination of *p* < 0.20. In the prediction model, age was tested multiple times: continuously using both linear and quadratic terms and dichotomously with thresholds of 40, 45 and 55 years for comparison with previous publications.^1,6,21^ Interaction was tested for combinations that were clinically the most plausible: suspected invasive component and DCIS grade, age <45 years (based on cut-off age in active surveillance) in combination with BI-RADS score, or palpability, or DCIS grade. To account for missing data, multiple imputation with fully conditional specification was used in the multivariable logistic analysis. Twenty imputed data sets were generated, and the results were pooled according to Rubin’s rules. Based on the imputed data, a formula was defined to predict the risk. Finally, internal validation of the model was performed with bootstrap repetitions (200 times). The logistic regression model was evaluated with the area under the curve (AUC) of the receiver operating characteristic (ROC). Based on the predicted risks, patients were divided into five subgroups, and the association with minimal-volume DCIS and unfavourable features was analysed with the *p*-trend test for proportions. The analyses were done with STATA statistics/data analysis, version 13.1, StataCorp, Texas and in R, with the rms package for the evaluation of the predictive performance and the mice package for multiple imputation.

## Results

Of 3281 lesions that were selected with a preoperative biopsy diagnosis DCIS, 64 (2.0%) were excluded because they were not registered in the NCR, and 15 (0.5%) because registration was incomplete. In addition, to answer the research question accurately, more were excluded: 60 (1.8%) that did primarily not undergo excision, 143 (4.4%) for which the biopsy diagnosis was inconclusive and 107 (3.3%) with an ipsilateral history of DCIS or invasive breast cancer, resulting in 2892 DCIS diagnoses included in the study. Of these, 379 (13%) had missing data for one or more potential risk factor: 148 for palpability, 223 for BI-RADS score, 84 for DCIS grade and 81 for detection mode.

Of the 2892 DCIS diagnoses at biopsy, 596 (20.6%) were underestimated, as the diagnosis was invasive breast cancer at excision. Table 2 shows patient and biopsy characteristics and their relation with underestimation. Of biopsy DCIS, 66% was screen detected, 22% was palpable, 13% had a BI-RADS score 3, 75% had a BI-RADS score 4, 12% had a BI-RADS score 5 and 5% had a suspected invasive component at biopsy. The DCIS grade distribution was 15% low, 39% intermediate and 46% high (*p* = 0.001). Of the intermediates, 13% were low to intermediate or consisted of both low-grade and intermediate-grade DCIS, 21% were intermediate to high grade or consisted of both intermediate-grade and high-grade DCIS. The underestimation rate was 21% on average for all cases, 26% for non-screen-detected lesions, 36% for palpable lesions, 41% for BI-RADS score 5 and 23% for high-grade DCIS (*p* values between different categories were <0.001 for all variables). The risk factors with the greatest differences in underestimation rate for subgroups were palpability, with a 20% higher rate for palpable than for non-palpable lesions, BI-RADS score, with a 25% higher rate for BI-RADS score 5 than for score 3, and suspected invasive component, with a 31% higher rate for suspected invasive component than for none. Of 596 invasive breast cancers, 47 were T1mi and 207 were T1a. The underestimation rate when filtering out all lesions of 5 mm or smaller was 11.8% (*n* = 342).

**Table 2:** Distribution of underestimation rate

	All	Underestimated invasive breast cancer				*p* value
		No		Yes		
	*N*	*N*	%	*N*	%	
Total	2892	2296	79.4%	596	20.6%	
Age (years), mean (range)	58.7 (24–91)	58.9 (30–88)		57.8 (24–91)		0.033
*Age categories*						<0.001
<45 years	207	142	69%	65	31%	
≥45 years	2685	2154	80%	531	20%	
*Detection mode*						<0.001
Screen detected	1850	1521	82%	329	18%	
Otherwise	961	714	74%	247	26%	
Missing	81	61	75%	20	25%	
*Palpable*						<0.001
No	2147	1794	84%	353	16%	
Yes	597	380	64%	217	36%	
Missing	148	122	82%	26	18%	
*BI-RADS score*						<0.001
3	365	306	84%	59	16%	
4	1996	1638	82%	358	18%	
5	308	183	59%	125	41%	
Missing	223	169	76%	54	24%	
*DCIS histological grade at biopsy*						0.001
Low	422	360	85%	62	15%	
Intermediate	1083	866	80%	217	20%	
High	1303	1006	77%	297	23%	
Missing	84	64	76%	20	24%	
*Suspected invasive component at biopsy*						<0.001
No	2743	2222	81%	521	19%	
Yes	149	74	50%	75	50%	
*Synchronous contralateral breast tumour*						0.181
No	2796	2225	80%	571	20%	
Yes	96	71	74%	25	26%	
*Preoperative MRI*						<0.001
No (or unknown)	2188	1773	81%	415	19%	
Yes	704	523	74%	181	26%	
*Preoperative multidisciplinary team meeting*						0.364
No (or unknown)	301	245	81%	56	19%	
Yes	2591	2051	79%	540	21%	
*First resection*						<0.001
Wide local excision	1822	1510	83%	312	17%	
Mastectomy	1070	786	73%	284	27%	

Table 3 shows the results of univariable and multivariable analysis of the risk for underestimation of preoperatively known potential risk factors for invasive breast cancer. Age and detection mode were statistically significant in univariable analysis, but not in multivariable analysis. Both were associated with palpability and BI-RADS score, and age was also associated with DCIS grade (shown in supplement 1, along with other associations). In multivariable analysis, grade, palpability, BI-RADS score and a suspected component were significant.

**Table 3:** Risk factors for underestimation

Preoperative patient and lesion characteristics^a^	Logistic regression analysis for underestimation of invasive breast cancer					
	Univariable			Multivariable^b^		
	OR	95% CI	*p* value	OR	95% CI	*p* value
*Age (years)*						not in the model^c^
<45	1.86	1.34–2.53	<0.001			
≥45	1					
*Detection mode*						
Screen detected	1			1		
Otherwise	1.60	1.33–1.93	<0.001	1.16	0.94–1.45	0.164
*Palpable*						
No	1			1		
Yes	2.90	2.37–3.55	<0.001	2.22	1.76–2.81	<0.001
*BI-RADS score*			<0.001			<0.001
3	0.88	0.65–1.19	0.487	0.86	0.64–1.17	0.348
4	1			1		
5	3.13	2.43–4.03	<0.001	2.36	1.80–3.09	<0.001
*DCIS histological grade at biopsy*			0.001			0.078
Low	1			1		
Intermediate	1.45	1.06–1.98	0.017	1.36	0.99–1.87	0.054
High	1.71	1.27–2.31	<0.001	1.43	1.05–1.95	0.025
*Suspected invasive component biopsy*						
No	1			1		
Yes	4.32	3.09–6.04	<0.001	3.84	2.69–5.46	<0.001

^a^For all interaction variables *p* > 0.05: grade and suspect, *p* = 0.469, age <45 years; palpable, *p* = 0.168, age <45 years; BI-RADS, *p* = 0.996, age <45 years; DCIS grade, *p* = 0.108

^b^Based on the imputed dataset

^c^Age: continuous: *p* = 0.552, quadratic relationship (adding a quadratic term): *p* = 0.257, dichotomous with threshold 40 years: *p* = 0.923, dichotomous with threshold 45 years: *p* = 0.421, dichotomous with threshold 55 years: *p* = 0.644

For each of the 2892 DCIS, the risk of an underestimated invasive breast cancer was calculated based on the prediction model with the following formula:

$${\mathrm{Predicted}}\,{\mathrm{risk}} = \left( {\frac{1}{{1 + {\mathrm{e}}^{ - {\mathrm{score}}}}}} \right) \times 100\% ,$$

with score = −2.1131 + 0.1555 × detection_mode_otherwise + 0.7985 × palpable − 0.1464 × BI-RADS_score_3 + 0.8589 × BI-RADS_score_5 + 0.3111 × intermediate_DCIS_grade + 0.3571 × high_DCIS_grade + 1.3445 × suspected_invasive_component, where for all risk factors: 1 = if condition applies, 0 = otherwise.

For example, the predicted risk is calculated as follows for a screen-detected DCIS which is non-palpable, has a BI-RADS score 4, an intermediate-grade and no suspected invasive component: score = −2.1131 + 0.1555 × 0 + 0.7985 × 0 − 0.1464 × 0 + 0.8589 × 0 + 0.3111 × 1 + 0.3571 × 0 + 1.3445 × 0 = −1.802, and

$${\mathrm{Predicted}}\,{\mathrm{risk}} = \left( {\frac{1}{{1 + {\mathrm{e}}^{ - {(- 1.802)}}}}}\right)\times 100\% = 14.2\%.$$

The risk for an individual patient can be calculated in a user-friendly way with a calculation tool in Evidencio, https://www.evidencio.com/models/show/1074.

The predicted risks ranged from 9.5 to 80.2%, the mean was 20.6% and the median was 14.7%. The predicted risk for underestimation was on average 27.4% for the biopsy of DCIS that were underestimated invasive breast cancers, whereas it was on average 18.8% for the biopsy of DCIS that also had the DCIS diagnosis at excision. The predicted risks for each combination of risk factors are shown in supplement 2. The matching of the predicted risks with the observed rate is shown in supplement 3.

**Fig. 1:**
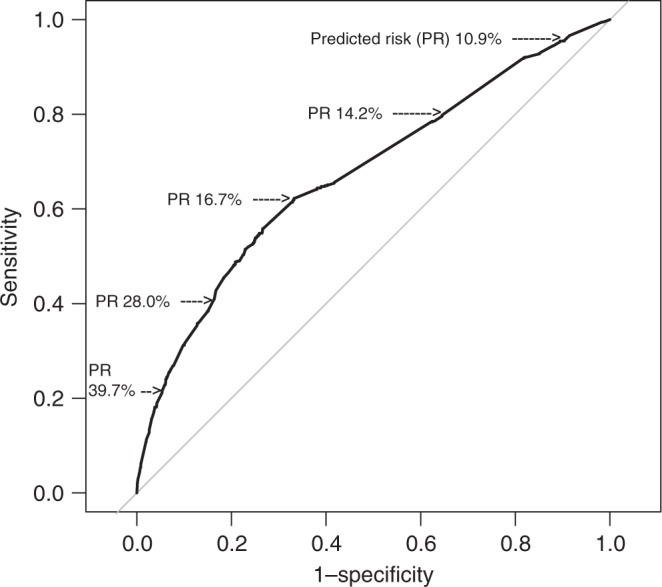
Performance of the model in relation to the chosen cut-off point of the predicted risks

The ability of the model to separate DCIS as diagnosis after excision from underestimated invasive breast cancer is shown in Fig. 1. To draw this figure, the DCIS were divided into low-risk or high-risk DCIS based on a cut-off point, and for each point the sensitivity and 1-specificty was calculated. In this study, the sensitivity means the rate of underestimated invasive breast cancer that was correctly predicted as high risk, and 1-specificity means the rate of DCIS at excision that was falsely predicted as high risk. The AUC (*c*-index) of the ROC was 0.668 and the AUC, corrected for optimism by bootstrapping, was 0.661. The AUC for a model based only on lesions >5 mm was 0.69.

Based on the predicted risks, the DCIS biopsies were divided into five subgroups; the characteristics of each subgroup are shown in Table 4. From the subgroups with the lowest predicted risk to the subgroup with the highest predicted risk, the underestimation rate increased from 10.7 to 40.1%.

**Table 4:** Risk groups according to percentile of the predicted risk^a^

	<2–20 percentile		2–<40 percentile		40–<60 percentile		60–<80 percentile		≥80 percentile		*p* value
Number of lesions	472		526		643		632		619		
Mean predicted risk (range)	11.6 (9.5–14.1)%		14.2 (14.2–14.7)%		14.8 (14.7–16.1)%		21.9 (16.2–28.0)%		39.1 (28.0–80.2)%		
Rate of underestimated invasive breast cancers (*n*)	10.4%	(49)	15.2%	(80)	13.2%	(85)	21.8%	(138)	39.4%	(244)	
Rate of invasive breast cancers with unfavourable features (*n*/total)	1.7%	(8/472)	4.0%	(21/526)	4.7%	(30/643)	9.0%	(57/632)	19.1%	(118/619)	*p*<0.001
Rate of minimal-volume DCIS, DCIS completely removed via biopsy (*n*/total)	18.4%	(87/472)	7.6%	(40/526)	4.2%	(27/643)	4.9%	(31/632)	1.8%	(11/619)	*p*<0.001

^a^For each DCIS, a predicted risk was calculated with the prediction model. Based on these risks, the DCIS were divided into five subgroups, with the percentile <20% comprising the 20% of DCIS with the lowest predicted risk, percentile ≥80% comprising the 20% of DCIS with the highest risk, etc

The associations between the predicted risks and minimal-volume DCIS were as follows: the rates of minimal-volume DCIS decreased from 18.0 to 1.6% from the subgroups with the lowest predicted risk to the subgroup with the highest predicted risk, *p* < 0.001 (see Table 4). On average, 6.8% of DCIS diagnoses at biopsy were minimal-volume DCIS, in which the DCIS was completely removed via biopsy (meaning 8.5% of the 2296 lesions with the DCIS diagnosis at excision).

The associations between the predicted risks and unfavourable features were as follows: the percentage of invasive breast cancers with unfavourable features increased from 15.9 to 49.5% from the lowest to the highest predicted risk group, *p* < 0.001 (see Table 4). On average, 39% of the invasive breast cancers had unfavourable features. More details on the distribution of tumour characteristics of the 596 invasive breast cancers are shown in supplement 4. Of the invasive breast cancers, 27% were grade III, 26% were Her2Neu positive, 8% were triple negative, 77% were TNM stage 1A (size at maximum 2.0 cm and no metastasis) and the median size was 6 mm.

## Discussion

The aim of our study was to expand the knowledge on underestimation of invasive breast cancer at core-needle biopsy in the routine clinical practice in the Netherlands and to develop a prediction model based on the analysis of a retrospective population-based dataset of 2892 DCIS diagnoses. We also analysed the association of predicted risk with minimal-volume DCIS and with the occurrence of unfavourable features of the underestimated invasive breast cancer.

The risk for underestimation of invasive breast cancer after a DCIS diagnosis was almost 21%. Preoperatively known risk factors for an underestimated diagnosis of invasive breast cancer were a high DCIS grade, a palpable tumour, a BI-RADS score 5 and a histologically suspected invasive component. Detection mode was also included in the model, although the association with underestimation was comparably weak. The predicted risk for underestimation ranged from 9.5 to 80.2%. Of the 596 underestimated invasive breast cancers, 39% had unfavourable features. Of the DCIS diagnoses at excisional pathology, 6.8% were minimal-volume DCIS.

The underestimation rate of 20.6% shows that excision of the DCIS is still not only important for preventing DCIS from progressing to invasive breast cancer but also for finding already existing invasive breast cancers. The rate found in our study was in between the 25.9% of a meta-analysis published in 2011 and the recently reported 14.1% of a large single institution study.^14,16^ The underestimation rate is associated with the diagnostic work-up whereby there is a tendency to decreasing underestimation rates in more recent time period. This study used data from 2011 and 2012. At that time vacuum-assisted biopsy was not yet commonly used in the Netherlands, therefore we assume that the underestimation rate currently will be somewhat lower in the Netherlands. And in the period 2011–2012, hospitals often used screen film mammography, but the screening mammography was already digitized, and therefore no major difference in underestimation rate. The Netherlands currently is assumed because of this change in technique.

This population-based study showed several clinical, radiological and pathological features that are all routinely available before operation as risk factors for underestimation.

The risk factors we found are partly similar to those reported in literature. Differences could be due to sample size, as this study was much larger than other studies: studies in literature had 172 to 834 cases and up to 145 events, whereas we had 2892 cases and 589 events. Differences in study outcomes could also be caused by the combination of available data and the correlation between many data. For age, others found various risks for the youngest age category: no increase^25^, increased but not significantly so^16^ and univariable significant but not in multivariable analysis^14,21^. In our study, young age was also only univariably associated with underestimation. For DCIS grade, the risk of underestimation for intermediate grade was in between the risk for low-grade and high-grade DCIS. This was also reported by some other studies^14,20,27^, whereas others reported the risk for intermediate-grade DCIS as comparable to that of the high-grade risk^19,25^. In our study the DCIS grade was less discriminative than the other risk factors in the model, but on the other hand the underestimation rate of 15% for low DCIS grade was the lowest rate for a subgroup in the model and high grade was the largest subgroup with an increased risk. Palpability of the lesion has consistently been reported as a risk factor, which this study could confirm.^15,16,18,19,22–24,26,37^ The BI-RADS score is an assessment categorization that should give an indication of the likelihood of cancer based on the interpretation of the radiologist. We showed that it is associated with the underestimation rate; the difference between BI-RADS score 4 and 5 was 23% in underestimation rate, which is much larger than the 7–8% found by others.^16,21^ A larger difference was reported in a study with a high average underestimation rate due to a high rate of micro-invasion.^15^ Still, the study of Kim et al.^15^ is interesting because they found a somewhat higher underestimation rate for BI-RADS score 4c, compared to 4a and 4b. It is worth noting that the BI-RADS score has not yet been investigated very extensively. A suspected invasive component has also only been reported in a limited number of studies.^23,24^; all found a high risk for underestimation for biopsies with a suspected component.

The prediction model we developed with the identified risk factors must be used wisely. For selecting high-risk lesions, it has to be noted that lesions with a high predicted risk still have a good chance of a final diagnosis of DCIS since the sensitivity of the model was low. The sensitivity or the AUC was higher in several other studies.^14,17,22,24,28^ Each study with a prediction model used different risk factors and therefore the models are not easily comparable. This has also been demonstrated in external validation of studies that applied published models to their cases; one study demonstrated a tendency towards lower or higher numbers of observed underestimates than expected^29^, and another previous study demonstrated validation AUCs of 0.59–0.66, whereas the studies they validated reported validities of 0.70–0.85^14^. The low AUC in this study could also be due to the absence of certain data that might have been important, such as the type of biopsy device and the size of the lesion on mammography. This is shown in Table 1, where the references that were made bold are the results of the studies making a prediction model, whereas the variable names that are given in bold are the variables that were analysed in this study.

Part of the DCIS was minimal-volume DCIS and was thus removed from the biopsy itself. In this study, minimal-volume DCIS was associated with the predicted underestimation risk. To our knowledge, this information has never been demonstrated before; one study demonstrated a similar rate of minimal-volume DCIS, but the association with underestimation was not investigated.^31^ In our study, the minimal-volume DCIS was higher for the predicted low-risk group.

The invasive breast tumours that were found at excision were heterogeneous in prognostic and predictive features. Underestimated invasive tumours are often small: the median size was 6 mm, which is in line with or somewhat lower than the results of other studies.^17,25–27^ On the other hand, 8% were TNM stage IIB or III, and 20% were triple negative or ER-PR-Her2Neu+. Where other studies analysed none or a few tumour characteristics, we had numerous tumour-related data of the 589 underestimates. Based on these data, we calculated the rate of cancers with unfavourable features, which was 39%. For these patients, systemic therapy was indicated. In our study, the rate of unfavourable features was higher for the predicted high-risk DCIS group.

Due to its retrospective nature, this study has certain limitations. A limitation in interpreting the results is that the preoperative decisions and techniques were not standardized, and therefore the preferences of the treating physicians and the patients will have influenced the underestimation results. For instance, for a high-grade DCIS with histological suspicion of invasiveness, the biopsy can be repeated (and invasive breast cancer might be found preoperatively) or initial treatment can be started (with an increased risk of underestimation). Also, for DCIS grade, other studies might have used different grading systems. Another limitation is that results of observational studies are difficult to compare because of differences in diagnostic work-up, differences in major selection criteria, such as the presence of micro-invasion, differences in investigated risk factors and associations between the investigated risk factors. Our dataset did not provide information on the number of biopsies nor on the biopsy device, and hence the amount of tumour taken at biopsy was not known. Some other factors were not available either, such as the presence of comedo-necrosis, the breast density, the visibility of the lesion on ultrasound, the presence of mammographic mass or the size of the lesion seen on the mammogram.

The model in this study is based on a large dataset that is based on nationwide Dutch data, and it demonstrated the association of risk for underestimation with minimal-volume DCIS and unfavourable features of invasive cancer, which makes the results valuable. The prediction model could be improved by adding additional data; the most interesting targets of investigation for future research are the biopsy type and mammography-related data: BI-RADS score 4 subcategories, the underlying reasons for a BI-RADS score (such as mass), size of the lesion and presence of residual mammographic abnormalities after biopsy. Furthermore, the prediction model should be validated externally.

## Conclusion

Our results demonstrated that the risk for an underestimated diagnosis of invasive breast cancer after a diagnosis of DCIS at biopsy is considerable. Of these invasive breast cancers, two-fifths has unfavourable features. With our prediction model, the individual risk of underestimation can be calculated based on routinely available preoperatively known risk factors

## Electronic supplementary material

Supplementary info 1 - associations risk factors

Supplementary info 2 - predicted risks

Supplementary info 3 - calibration plot

Supplementary info 4 - tumour characteristics

## Data Availability

The dataset generated for this current study are not publicly available due additional research questions to be answered, but is available from the corresponding author on reasonable request. The prediction model is available for external validation via Evidencio (model 1074).
