# Supplementary material for: A prediction model for underestimation of invasive breast cancer after a biopsy diagnosis of ductal carcinoma in situ: based on 2892 biopsies and 589 invasive cancers
Source: Br J Cancer. 2018 Oct 17;119(9):1155–62. doi: 10.1038/s41416-018-0276-6 (PMC6219477; doi:10.1038/s41416-018-0276-6)
Supplement: Supplementary file 1 — Supplementary info 1 - associations risk factors [file 41416_2018_276_MOESM1_ESM.pdf]

Supplement 1: Associations between risk factors

Of 2892 DCIS included in the study, 2513 had no missing data for one or more potential risk factor.

For these the associations between risk factors are shown in the table. The percentages in the table are row percentages.

For overview purposes not all values of a category are shown in the columns; the percentages of the values ≥ 45 years, detection mode otherwise, non-palpable and no suspected invasive component can be deducted from the other values. For example, there were 175 patients that were < 45 years, the DCIS of 60% was palpable and of 40% non-palpable.

Age and detection mode were both associated with palpability and BI-RADS score, and age was also associated with DCIS grade.

	Number	%	p-value	%	p-value	%	p-value	%	%	%	p-value	%	%	%	p-value	%	p-value
		Age		Detection mode		Palpable		BI-RADS score				DCIS grade at biopsy				Suspected invasive component	
		<45 years		Screen-detected		Yes		3	4	5		Low	Inter-mediate	High		Yes	
Age			x		<0.001		<0.001				<0.001				0.012		0.857[$]
< 45 years	175	x		0%		60%		23%	58%	18%		10%	33%	57%		5%	
>=45 years	2338	x		72%		18%		13%	76%	11%		15%	39%	46%		5%	
Detection mode			<0.001 [$]		x		<0.001				<0.001				0.939		0.327
Screening	1689	0%		x		10%		11%	80%	10%		15%	39%	46%		5%	
Otherwise	824	21%		x		43%		20%	64%	15%		15%	38%	47%		6%	
Palpable			<0.001		<0.001		x				<0.001				0.760		<0.001
No	1990	4%		76%		x		14%	78%	8%		15%	39%	46%		4%	
Yes	523	20%		33%		x		14%	60%	26%		14%	38%	48%		8%	
BI-RADS score			<0.001		<0.001		<0.001				x				<0.001		0.079
3	349	12%		52%		20%		x	x	x		24%	42%	34%		4%	
4	1874	5%		72%		17%		x	x	x		14%	38%	48%		5%	
5	290	11%		56%		47%		x	x	x		9%	35%	56%		8%	
DCIS grade at biopsy			0.012		0.939		0.760				<0.001				x		<0.001[$]
Low	371	5%		67%		20%		22%	71%	7%		x	x	x		2%	
Intermediate	971	6%		68%		20%		15%	74%	11%		x	x	x		3%	
High	1171	9%		67%		21%		10%	76%	14%		x	x	x		7%	
Suspected invasive component			0.857 [$]		0.327		<0.001				0.079				<0.001 [$]		x
No	2388	7%		67%		20%		14%	75%	11%		15%	39%	45%		x	
Yes	125	7%		63%		34%		11%	71%	18%		5%	26%	70%		x	

[$]: Fisher's exact test. Other associations were tested with the Pearson chi-square test.
