# Supplementary material for: A prediction model for underestimation of invasive breast cancer after a biopsy diagnosis of ductal carcinoma in situ: based on 2892 biopsies and 589 invasive cancers
Source: Br J Cancer. 2018 Oct 17;119(9):1155–62. doi: 10.1038/s41416-018-0276-6 (PMC6219477; doi:10.1038/s41416-018-0276-6)
Supplement: Supplementary file 2 — Supplementary info 2 - predicted risks [file 41416_2018_276_MOESM2_ESM.pdf]

Supplement 2: Predicted risk for each combination of risk factors

Of 2892 DCIS included in the study, 2513 had no missing data for one or more potential risk factor.

For these, the combination of risk factors is shown in the table along with the size of the group, the predicted risk and the percentile group. Highlighted in colour are the combinations with the highest number of DCIS;

number of DCIS: 50 - <100

number of DCIS: ≥ 100

Based on the predicted risk, the DCIS were grouped in one of the five percentile groups. The group <20% comprises the 20% of DCIS with the lowest predicted risk, percentile ≥ 80% comprises the 20% of DCIS with the highest risk, etc. The predicted risks on average per percentile groups were 11.6%, 14.2%, 14.8%, 21.9% and 39.1%.

In the percentile group with the lowest risk, all DCIS were non-palpable and had no suspected invasive component at biopsy. DCIS with a suspected invasive component were all in the highest percentile group.

Percentile group	Predicted risk (%)	Number of DCIS	Detection mode	Palpable (before biopsy)	BI-RADS score	DCIS grade at biopsy	Suspected invasive component at biopsy
<20	9.45	35	screen-detected	no	3	low	no
	10.78	173	screen-detected	no	4	low	no
	10.87	30	otherwise	no	3	low	no
	12.37	38	otherwise	no	4	low	no
	12.47	73	screen-detected	no	3	intermediate	no
	12.98	61	screen-detected	no	3	high	no
20 - <40	14.16	461	screen-detected	no	4	intermediate	no
	14.27	42	otherwise	no	3	intermediate	no
40 - <60	14.73	537	screen-detected	no	4	high	no
	14.84	27	otherwise	no	3	high	no
60 - <80	16.16	114	otherwise	no	4	intermediate	no
	16.79	170	otherwise	no	4	high	no
	18.83	2	screen-detected	yes	3	low	no
	21.17	21	screen-detected	yes	4	low	no
	21.32	15	otherwise	yes	3	low	no
	22.20	11	screen-detected	no	5	low	no
	23.88	26	otherwise	yes	4	low	no
	24.05	2	screen-detected	yes	3	intermediate	no
	24.90	4	screen-detected	yes	3	high	no
	25.00	6	otherwise	no	5	low	no
	26.82	36	screen-detected	yes	4	intermediate	no
	27.00	27	otherwise	yes	3	intermediate	no
	27.74	51	screen-detected	yes	4	high	no
	27.92	17	otherwise	yes	3	high	no
>80	28.03	42	screen-detected	no	5	intermediate	no
	28.97	57	screen-detected	no	5	high	no
	29.98	85	otherwise	yes	4	intermediate	no
	30.96	73	otherwise	yes	4	high	no
	31.27	16	otherwise	no	5	intermediate	no
	31.68	3	screen-detected	no	4	low	yes
	31.88	1	screen-detected	no	3	low	yes
	32.27	14	otherwise	no	5	high	no
	35.35	2	screen-detected	no	3	intermediate	yes
	36.40	4	screen-detected	no	3	high	yes

Percentile group	Predicted risk (%)	Number of DCIS	Detection mode	Palpable (before biopsy)	BI-RADS score	DCIS grade at biopsy	Suspected invasive component at biopsy
	38.76	18	screen-detected	no	4	intermediate	yes
	38.80	2	screen-detected	yes	5	high	no
	38.97	1	otherwise	no	3	intermediate	yes
	39.85	35	screen-detected	no	4	high	yes
	40.07	3	otherwise	no	3	high	yes
	42.51	2	otherwise	no	4	intermediate	yes
	42.55	6	otherwise	yes	5	low	no
	43.63	7	otherwise	no	4	high	yes
	46.39	18	screen-detected	yes	5	intermediate	no
	47.54	24	screen-detected	yes	5	high	no
	50.27	23	otherwise	yes	5	intermediate	no
	51.42	49	otherwise	yes	5	high	no
	54.62	2	otherwise	yes	4	low	yes
	55.99	1	screen-detected	yes	3	high	yes
	58.44	2	screen-detected	yes	4	intermediate	yes
	58.66	1	otherwise	yes	3	intermediate	yes
	59.55	6	screen-detected	yes	4	high	yes
	59.77	1	otherwise	yes	3	high	yes
	59.90	2	screen-detected	no	5	intermediate	yes
	61.00	3	screen-detected	no	5	high	yes
	62.16	2	otherwise	yes	4	intermediate	yes
	63.24	12	otherwise	yes	4	high	yes
	64.63	2	otherwise	no	5	high	yes
	77.66	3	screen-detected	yes	5	high	yes
	79.50	2	otherwise	yes	5	intermediate	yes
	80.24	10	otherwise	yes	5	high	yes
