# Supplementary material for: A prediction model for underestimation of invasive breast cancer after a biopsy diagnosis of ductal carcinoma in situ: based on 2892 biopsies and 589 invasive cancers
Source: Br J Cancer. 2018 Oct 17;119(9):1155–62. doi: 10.1038/s41416-018-0276-6 (PMC6219477; doi:10.1038/s41416-018-0276-6)

Supplement 3: Calibration plot of the prediction model

Of 2892 DCIS included in the study, 379 (13%) had missing data for one or more potential risk factors. These missing data were accounted for via multiple imputations (20 times). For one of these imputed datasets the calibration plot was drawn.

The predicted risk for an underestimated invasive breast cancer versus the observed rate is plotted in the figure below. The distance between the grouped observations and the 45 degree line is a measure of the error of the prediction model. The statistics for the plot for this imputed dataset are: c-statistic (ROC) of 0.666, R^2 of 0.110, Intercept of -0.019, slope of 0.985.

The histogram of the x-axis reflects the frequency of a predicted risk. 9.0% of DCIS have a risk of <12.0%, 54.6% of DCIS have a predicted risk of <15.0% and 26.0% have a risk of >25.0%.

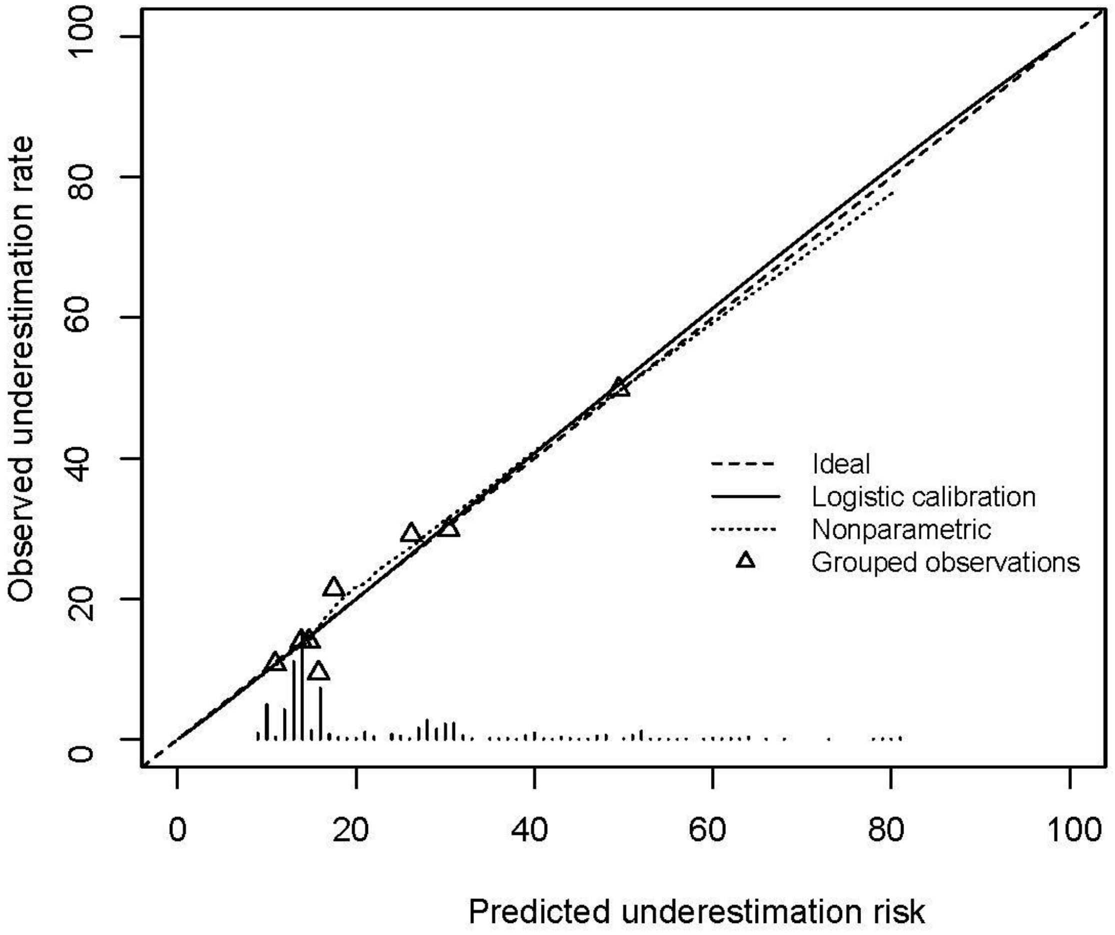

Supplement: Supplementary file 3 — Supplementary info 3 - calibration plot [file 41416_2018_276_MOESM3_ESM.pdf]
