# Supplementary material for: A prediction model for underestimation of invasive breast cancer after a biopsy diagnosis of ductal carcinoma in situ: based on 2892 biopsies and 589 invasive cancers
Source: Br J Cancer. 2018 Oct 17;119(9):1155–62. doi: 10.1038/s41416-018-0276-6 (PMC6219477; doi:10.1038/s41416-018-0276-6)
Supplement: Supplementary file 4 — Supplementary info 4 - tumour characteristics [file 41416_2018_276_MOESM4_ESM.pdf]

## Supplement 4: Predicted risk for each combination of risk factors

Of 2892 DCIS diagnoses at biopsy, 596 were underestimated invasive breast cancers.

Below are the tumour characteristics, based on the excisional specimens of these 596 cancers.

| | N | % |
|---|---|---|
| Morphology | 596 | |
| Lobular | 14 | 2% |
| Ductal | 531 | 89% |
| Mixed Ductal and Lobular | 29 | 5% |
| Other | 22 | 4% |
| Grade of the invasive tumour | 534 | |
| I | 165 | 31% |
| II | 225 | 42% |
| III | 144 | 27% |
| ER receptor | 542 | |
| Negative | 106 | 20% |
| Positive | 436 | 80% |
| PR receptor | 542 | |
| Negative | 206 | 38% |
| Positive | 336 | 62% |
| Her2Neu | 524 | |
| Negative | 386 | 74% |
| Positive | 138 | 26% |
| Receptor combinations | 520 | |
| ER - PR - Her2Neu - | 39 | 8% |
| ER + Her2Neu - | 343 | 66% |
| ER - PR - Her2Neu + | 61 | 12% |
| ER + Her2Neu + | 75 | 14% |
| ER - PR + | 2 | <1% |
| Tumour size (in mm) | 570 | |
| mean - median (range) | 9.5 - 6 ( 0 - 90) | |
| TNM stage | 596 | |
| I A | 460 | 77% |
| I B | 16 | 3% |
| II A | 73 | 12% |
| II B | 22 | 4% |
| III A | 16 | 3% |
| III B | 0 | 0% |
| III C | 9 | 1% |
| IV | 0 | 0% |
